# HTLV-1 proviral load as an indicative marker of HAM/TSP: a systematic review of studies of patients with HAM/TSP

**DOI:** 10.1186/1742-4690-11-S1-P36

**Published:** 2014-01-07

**Authors:** Shiva Shaan Bassi, Raimundo Coutinho, Viviana Nilla Olavarria, Fabiola Martin, Bernardo Galvão-Castro, Maria Fernanda Rios Grassi

**Affiliations:** 1 Advanced Laboratory of Public Health, Gonçalo Moniz Center, Fundação Oswaldo Cruz, Salvador, Bahia, Brazil; 2 Biology Graduate, Centre for Immunology and Infection, Department of Biology, University of York, UK; 3 Centre for Immunology and Infection, Department of Biology, Hull and York Medical School, University of York, York, UK; 4 Bahiana School of Medicine and Public Health (EBMSP) Salvador, Bahia, Brazil

## 

The diagnosis of HAM/TSP is based on criteria, as proposed by the World Health Organization, revised in 1989. This primarily relies on diagnosis through laboratory criteria and an extensive list of neurological signs and symptoms. Consequentially HAM/TSP diagnosis is often delayed or not confirmed, especially in countries with limited resources. There is strong evidence that high levels of proviral load (PVL) are more frequently found in HAM/TSP patients compared to asymptomatic carriers. Considering PVL as an additional diagnostic criterion for HAM/TSP diagnosis may be useful in classifying the disease at an earlier stage. However, there is yet to be a consensus on a PVL threshold for differentiating HAM/TSP and asymptomatic individuals. To further understand this critical cut-off point, we have systematically searched scientific databases and analysed data on HTLV-1 proviral loads in HAM/TSP patients compared to asymptomatic carriers. Median PVL data has been extracted from a multitude of cohort studies from both endemic and non-endemic countries. In addition, we have evaluated the methodologies used to measure proviral load, such as the amplified region of the HTLV-1 genome, DNA sample source and number of cells quantified. The analysis of these results may help to determine a PVL that will distinguish HAM/TSP patients from asymptomatic individuals. Observations on this review, gives proposition to the need for an international, cross-sectional study with standardized methodologies that would provide a universal threshold that would finally enable clinicians to distinguish HTLV-1 infection at the different clinical stages through the use of a quantitative marker.

